# Percentages of Vaccination Coverage Required to Establish Herd Immunity against SARS-CoV-2

**DOI:** 10.3390/vaccines10050736

**Published:** 2022-05-08

**Authors:** Pedro Plans-Rubió

**Affiliations:** 1 Public Health Agency of Catalonia, Department of Health of Catalonia, 08005 Barcelona, Spain; pedro.plans@gencat.cat; 2 Ciber of Epidemiology and Public Health (CIBERESP), 28028 Madrid, Spain

**Keywords:** COVID-19 vaccination coverage, anti-SARS-CoV-2 herd immunity, COVID-19 vaccination strategy, SARS-CoV-2

## Abstract

The pandemic associated with SARS-CoV-2 is a worldwide public health challenge. The WHO has proposed to achieve 70% COVID-19 vaccination coverage in all countries by mid-2022. Nevertheless, the prevention strategy based on COVID-19 vaccination and other applied prevention measures has not been sufficient to prevent SARS-CoV-2 epidemic waves. This study assessed the vaccination coverage that would be required to establish herd immunity against SARS-CoV-2, taking into account virus transmissibility (R_o_ values from 1.1 to 10) and COVID-19 vaccination effectiveness. The study found that high percentages of vaccination coverage and high levels of vaccination effectiveness are necessary to block the transmission of Omicron and other SARS-CoV-2 variants with greater infectious capacity. COVID-19 vaccination programs could establish herd immunity against SARS-CoV-2, with R_o_ values ranging from 3 to 10 and levels of COVID-19 vaccination effectiveness of 70–100%. Factors reducing COVID-19 vaccination effectiveness (emergent variants, infections among vaccinated individuals, high risk individuals) and factors increasing SARS-CoV-2 transmissibility (close settings) increased the percentages of vaccination coverage that would be required to establish herd immunity. Two measures should be implemented to establish herd immunity against SARS-CoV-2: (1) achieve ≥ 90% COVID-19 vaccination coverage in all countries worldwide, and (2) increase the effectiveness of COVID-19 vaccines in preventing Omicron infection to at least 88%.

## 1. Introduction

The World Health Organization (WHO), on 11 March, 2020, declared the coronavirus disease 2019 (COVID-19) epidemic caused by severe acute respiratory syndrome coronavirus 2 (SARS-CoV-2) to be a global pandemic [1]. This pandemic dates back to December 2019, when a cluster of unexplained pneumonia cases was identified in Wuhan (China) [2]. Despite the global efforts that have been made during the last two years to mitigate the pandemic, SARS-CoV-2 continues to spread around the world. Globally, as of 18 April 2022, 500.2 million laboratory confirmed cases of SARS-CoV-2 infection have been reported, resulting in 6.2 million deaths [3]. The number of cases reported in last 24 hours was 948,845 [3].

The pandemic associated with SARS-CoV-2 is a worldwide public health challenge. The prevention strategy implemented to reduce the transmission of SARS-CoV-2 and mitigate the pandemic’s health and economic effects included: (1) measures to detect infected individuals, (2) measures to treat COVID-19, (3) measures to prevent SARS-CoV-2 infections and COVID-19, and (4) measures to reduce exposition to SARS-CoV-2. 

Vaccination is the primary method for preventing and controlling the SARS-CoV-2 pandemic. COVID-19 vaccination programs can reduce the incidence and mortality from SARS-CoV-2 infections by means of individual protection in vaccinated individuals and herd immunity protection in unvaccinated individuals. As of 10 April 2022, 11.37 million doses had been administered in the world, 58.2% of the world’s population had completed COVID-19 vaccination, and 64.7% had received at least one dose of the vaccine [4]. The coverage for completed vaccination was 58.2% in the World, 73.2% in South America, 67.7% in Asia, 65.3% in Europe, 62.9% in Oceania, 62.7% in North America, and 15.4% in Africa [4]. The percentages of full vaccination coverage range from 0.1% in Burundi to 96.3% in the United Arab Emirates [4] (Supplementary Materials Table S1). 

On May 2022, nine vaccines had been validated for use by the WHO after receiving emergency use authorizations: Pfizer/BioNTech, Moderna, AstraZeneca, Janssen, Sinopharm BIBP, Sinovac, CoronaVac, Covaxin, and Novavax [5] (Table 1). Five vaccines have been authorized by the European Medicines Agency (EMA): Pfizer/BioNTech, Moderna, AstraZeneca, Janssen, and Novavax [5]. Three vaccines have been authorized by the Food and Drug Administration of the United States (FDA): Pfizer/BioNTech, Moderna, and Janssen [5]. Five vaccines are under assessment by the WHO: Sputnik, Sinopharm WIBP, Convidecia, Sanofi–GSK, and SCB-2019 [5]. There are more than 300 vaccine candidates, 143 vaccines in different clinical stages of development, and 195 vaccines undergoing pre-clinical development [6].

The available COVID-19 vaccines have been developed using mRNA, virus vectors, recombinant protein subunit particles, and inactivated virus [5]. RNA vaccines contain mRNA, produced in vitro using viral sequences, that directs cells to produce copies of viral proteins. Virus vector vaccines contain a viral vector that introduces genetic material from the virus. Protein subunit vaccines contain harmless pieces (proteins) of the virus. Inactivated virus vaccines contain harmless inactivated virus. 

COVID-19 vaccines are associated with high anti-SARS-CoV-2 immunity levels and reductions in morbidity and mortality from COVID-19 [7,8]. The effectiveness of available vaccines in preventing severe disease ranged from 37% to 73% for the Omicron variant and from 49% to 97% for the Delta variant, and the effectiveness in preventing infections ranged from 49% to 97% for the Omicron variant and from 46% to 92% for the Delta variant [8] (Table 1). 

The WHO has proposed to achieve 70% COVID-19 vaccination coverage in all countries by mid-2022 [9]. It was expected that achieving 60–90% vaccination coverage could generate sufficient herd immunity to block the transmission of SARS-CoV-2 in the population [10]. Nevertheless, in April 2022, the full vaccination coverage was at least 70% in 27% of the countries of the world, and the intensive prevention measures implemented since 2020 have not been sufficient to prevent SARS-CoV-2 epidemic waves [4].

Several factors could explain the persistence of SARS-CoV-2 in the world. First, the percentages of vaccination coverage achieved could be insufficient to block the transmission of SARS-CoV-2 in the community and in different population groups. Second, the emergence of SARS-CoV-2 variants could escape the vaccine-induced immunity protection and natural immunity protection generated by previous variants. Recent studies suggest that the spread of the Omicron variant is associated with lower COVID-19 vaccination effectiveness [11,12]. Third, waning vaccine-induced immunity could make it difficult to reach and maintain herd immunity in the population [13,14]. Fourth, the lack of sufficient vaccination coverage among children and infants could be unable to prevent the transmission of SARS-CoV-2 infections to children and infants [15,16]. 

The questions are therefore: What are the percentages of COVID-19 vaccination coverage required to establish herd immunity against different SARS-CoV-2 in the population? Is the COVID-19 vaccination coverage objective of 70% sufficient to establish herd immunity against SARS-CoV-2? What are the effects of infections among vaccinated individuals and waning immunity protection on the percentages of vaccination coverage that would be required to establish herd immunity? 

The objectives of this study were: (1) to determine the percentages of COVID-19 vaccination coverage required to establish herd immunity against different SARS-CoV-2; (2) to assess the effect of Omicron-associated reinfections among vaccinated individuals on the percentages of vaccination coverage required to establish herd immunity; (3) to assess whether the WHO COVID-19 vaccination coverage objective of 70% and alternative objectives of 80% and 90% are sufficient to establish herd immunity against different SARS-CoV-2; (4) to assess the vaccination coverage required to establish herd immunity in populations with individuals already protected against SARS-CoV-2 due to natural infection. 

## 2. Materials and Methods

### 2.1. Herd Immunity against SARS-CoV-2

Herd immunity against SARS-CoV-2 viruses is defined as the indirect protection of susceptible individuals generated by a prevalence of protected individuals (I). Herd immunity is established in a population when the prevalence of immune or protected individuals is higher than the critical prevalence “herd immunity threshold” (I > I_c_) [17,18,19,20,21]. When this occurs, the transmission of SARS-CoV-2 viruses is blocked because the number of susceptible individuals infected per case is lower than 1. By contrast, when the prevalence of protected individuals is lower than the herd immunity threshold, the number of susceptible individuals infected per case is higher than 1, and infections grow exponentially, spreading the coronavirus disease in the community. For this reason, the objective of COVID-19 vaccination programs should be to achieve and maintain a prevalence of protected individuals in the community higher than the critical prevalence [10].

The establishment of herd immunity against SARS-CoV-2 in a community depends on community and viral factors. The transmissibility of SARS-CoV-2 can be measured by the basic reproductive number, R_o_, which indicates the number of secondary cases generated by one primary case in a totally susceptible population. The basic reproduction number R_o_ is a key parameter explaining COVID-19 epidemic dynamics because the herd immunity threshold depends on the virus-specific basic reproductive number R_o_. 

In a totally susceptible population, each infected case will generate R_o_ new cases, but the prevalence of susceptible individuals will decrease over time, reducing the R_o_ value to an effective basic reproduction number, R, depending on the prevalence of susceptible individuals (S) and the prevalence of protected individuals (I):

R = R_o_ S = R_o_ (1 − I). (1)

The herd immunity threshold in terms of the critical prevalence of protected individuals required to establish herd immunity against SARS-CoV-2 can be determined using the formula:

I_c_ = 1 − (1/R_o_). (2)

In this study, the prevalence of protected individuals required to establish herd immunity against SARS-CoV-2 was determined assuming that SARS-CoV-2 can be associated with basic reproduction numbers R_o_ ranging from 1.1 to 10 [22,23,24,25,26].

The transmissibility of SARS-CoV-2 measured by R_o_ values has increased since the beginning of the pandemic in China. R_o_ values ranging from 0.32 to 6.47 were obtained on January–April 2020, with R_o_ values ranging from 3.6 to 4.5 in Wuhan city, from 0.47 to 6.47 in different regions of China, and from 1.27 to 5 in different countries [22]. R_o_ values ranging from 1.4 to 6.49, with a mean of 3.28, were obtained in a review of articles published on January–February 2020 [23]. Recent analysis has obtained R_o_ values ranging from 3.2 to 8, with a mean of 5.08, for the Delta variant [24]. A study carried out in Denmark found that transmissibility could be 3.19 (95% CI: 2.82–3.61) times greater for Omicron than for Delta infections under the same epidemiological conditions [25,26]. It is possible to assume that the R_o_ values of SARS-CoV-2 ranged from 1.1 to 5 during 2020 and from 3 to 10 since 2020.

### 2.2. Vaccination Coverage Required to Establish Herd Immunity against SARS-CoV-2 

COVID-19 vaccination programs can establish herd immunity when the prevalence of vaccine-induced protected individuals is equal to or higher than the herd immunity threshold (I_v_ ≥ I_c_) [20,21]. In this situation, the average number of infected individuals generated per each infected case is lower than 1, and transmission of SARS-CoV-2 is blocked in the population. 

COVID-19 vaccination programs establish anti-SARS-CoV-2 herd immunity when the prevalence of vaccine-induced protected individuals, which depends on the vaccination coverage and vaccine effectiveness, is at least equal to the critical prevalence I_c_. For this reason, the critical vaccination coverage (V_c_) required to establish herd immunity against SARS-CoV-2 was determined by dividing the herd immunity threshold (I_c_) by the level of vaccine effectiveness (E):

V_c_ = I_c_/E = [1 − (1/R_o_)]/E. (3)

This method is based on the following assumptions: (1) homogeneous mixing of individuals within the population, (2) homogeneous distribution of infected and vaccine-induced protected individuals within the population, and (3) fully susceptible population [17,18,19,20,21].

In this study, the critical vaccination coverage was determined using Formula (3) for SARS-CoV-2, with R_o_ values ranging from 1.1 to 10 and levels of vaccination effectiveness in preventing SARS-CoV-2 infections ranging from 10% to 100%.

The available COVID-19 vaccines are effective in preventing SARS-CoV-2 infections, severe disease, and mortality from COVID-19 [7,8,27,28,29]. The effectiveness of COVID-19 vaccines in preventing SARS-CoV-2 infection ranged from 24% to 48% for Omicron infection and from 46% to 91% for Delta infection, the effectiveness in preventing severe Omicron disease ranged from 48% to 73%, and the effectiveness in preventing severe Delta disease ranged from 49% to 95% [8] (Table 1). 

The percentages of vaccination coverage that would be required to establish herd immunity are higher in close settings (schools, nursing homes, and health care centers) than in the general population due to 75% higher basic reproduction numbers (1.75 × R_o_) than in the general population setting [30,31]. In places with high-risk individuals (high-risk adults, elderly people, and children aged 2–14 years), the required vaccination coverage can be higher than in the general population because vaccination effectiveness can be 20% lower (0.80 × effectiveness) than in healthy adults [31,32].

### 2.3. Vaccination Coverage Required to Establish Herd Immunity against SARS-CoV-2 with Infections among Vaccinated Individuals

The critical vaccination coverage required to establish herd immunity should be higher if infections occur among vaccinated individuals. All available vaccines are effective against severe illness, hospitalizations, and deaths, but recent studies have found that fully vaccinated people who become infected with the Omicron variant can spread the virus to other individuals [11,28].

When infections occur among vaccinated individuals, the critical prevalence of protected individuals associated with herd immunity should be higher than without infections, because infected vaccinated individuals should be considered as unprotected in herd immunity calculations. If r is the proportion of infections among vaccinated individuals, the prevalence of infected vaccinated individuals is equal to the vaccination coverage multiplied by the proportion of infections among them (V × r).

The critical vaccination coverage (V_c_) required to establish herd immunity was adjusted for infections among vaccinated individuals by taking into account the fact that infected vaccinated individuals increased the prevalence of vaccine-induced protected individuals necessary to establish herd immunity by “V_c_ × r”:

I_c_ = I_c_ + (V_c_ × r). (4)

In this study, the following formula derived from Equations (3) and (4) was used to determine the critical vaccination coverage (V_c_) that would be required to establish herd immunity, adjusted for infections among vaccinated individuals:

V_c_ = I_c_ /(E − r). (5)

The critical percentages of vaccination coverage required to establish herd immunity, adjusted for infections among vaccinated individuals, were determined for SARS-CoV-2, with R_o_ values ranging from 1.1 to 10, levels of vaccination effectiveness ranging from 10% to 100%, and the proportion of infections among vaccinated individuals at 5% and 9.8% [11]. 

Before the spread of Omicron variant, reinfections were very infrequent among infected individuals, with reinfection rates of 0–1.1% [33]. Prior to Omicron, SARS-CoV-2 infections were associated with a relative risk of reinfection of 0.15 for 6 months compared with those without prior infection [34]. Nevertheless, the Omicron variant is associated with a higher risk of reinfections than previous variants [11,28,35]. A study carried out in the United Kingdom including 116,683 individuals identified with an Omicron infection found a 9.8% proportion of SARS-CoV-2 reinfections [11]. Another study carried out in the United Kingdom using conditional Poisson regression to predict reinfection status (controlled for vaccine status, age, sex, ethnicity, asymptomatic status, region, and specimen date) found that Omicron was associated with a 5.41 higher risk of reinfection compared with Delta [28]. The risk of reinfection with Omicron was 5.02 higher in vaccinated individuals and 6.38 higher in unvaccinated individuals compared with Delta [28]. A study carried out in South Africa found a 2.39 higher risk of reinfection for the Omicron variant [35]. 

Infections among vaccinated individuals can be explained by waning anti-SARS-CoV-2 vaccine-induced immunity over time. SARS-CoV-2 infections among vaccinated individuals under endemic conditions could likely occur between 6 months and 5 years after peak antibody response [36,37]. Assuming that vaccine-induced protection is similar to natural protection after SARS-CoV-2 infection, it is possible to assume that a proportion of infections ranging from 5% or 9.5% could occur among vaccinated individuals. 

### 2.4. Assessment of Herd Immunity Levels Achieved with Percentages of Vaccination Coverage of 70%, 80%, and 90%

The WHO has proposed to achieve an overall COVID-19 vaccination coverage of 70% [9]. In this study, the model was used to assess whether the WHO COVID-19 vaccination coverage objective of 70% and alternative objectives of 80% and 90% could be sufficient to establish herd immunity against different SARS-CoV-2.

The vaccination coverage objective of 70% (or alternative objectives of 80% and 90%) was considered sufficient to establish herd immunity against SARS-CoV-2 when the vaccination coverage of 70% (or 80% and 90%) was equal to or lower than the critical vaccination coverage (V_70_ ≤ V_c_) that would be required for different levels of vaccination effectiveness. In this situation, the prevalence of vaccine-induced protected individuals achieved with COVID-19 vaccination is equal to or higher than the critical prevalence of protected individuals blocking SARS-CoV-2 transmission (I_v_ ≥ I_c_). 

Herd immunity levels associated with the COVID-19 vaccination objectives of 70%, 80%, and 90% were determined for populations without and with 9.8% of reinfections among vaccinated individuals. 

The R_o_ values of SARS-CoV-2 that COVID-19 vaccination programs could block by achieving 70%, 80%, and 90% vaccination coverage were determined using the formula:

R_o_ = 1/[1 − (V × E)]. (6)

The R_o_ values of SARS-CoV-2 that COVID-19 vaccination programs could block by establishing herd immunity with 70%, 80%, and 90% vaccination coverage depend on the prevalence of vaccine-induced protected individuals blocking SARS-CoV-2 transmission with these percentages of vaccination coverage. This prevalence was determined from the vaccination coverage (70%, 80%, and 90%) and vaccination effectiveness in preventing SARS-CoV-2 infection (presented in Table 1) (I_v_ = V × E). In this situation, the prevalence of vaccine-induced protected individuals is equal to the critical prevalence of protected individuals associated with herd immunity (I_v_ = I_c_) against SARS-CoV-2. COVID-19 vaccination programs could not block the transmission of viruses with R_o_ values associated with herd immunity thresholds higher than the prevalence of vaccine-induced protected individuals generated with 70%, 80%, and 90% vaccination coverage (I_c_ > V × E).

### 2.5. Vaccination Coverage Required to Establish Herd Immunity in Populations with Part of the Population Already Protected against SARS-CoV-2 

The vaccination coverage that would be required to establish herd immunity will be lower when part of the population is already protected against COVID-19 due to natural infections (I_n)_. In this situation, the prevalence of vaccine-induced protected individuals (I_v_) that would be required to establish herd immunity is lower depending on the prevalence of individuals already protected (I_v_ = I_c_ − I_n_). In this study, the vaccination coverage that would be required to establish herd immunity in communities when part of the population is already protected against COVID-19 due to natural infections was estimated using the following formula:

V_c_ = (I_c_ − I_n_)/E. (7)

In this formula, I_c_ is the prevalence of protected individuals required to establish herd immunity (herd immunity threshold), I_n_ is the prevalence individuals already protected against SARS-CoV-2 due to natural infections, and E is the level of COVID-19 vaccination effectiveness.

The required percentages of vaccination coverage were determined for communities with a prevalence protected individuals of 10% and 20%, R_o_ values for SARS-CoV-2 ranging from 1.1 to 10, and levels of COVID-19 vaccination effectiveness ranging from 10% to 100%.

### 2.6. Statistical Analysis

Microsoft Excel 2016 (v. 2203) was used to calculate the percentages of vaccination coverage that would be required to establish herd immunity against SARS-CoV-2 for R_o_ values ranging from 1.1 to 10, vaccination effectiveness from 10% to 100%, and 0%, 5%, or 9.8% of infections among vaccinated individuals.

Microsoft Excel 2016 (v. 2203) was used to estimate the levels of COVID-19 vaccination effectiveness required to establish herd immunity against SARS-CoV-2 (R_o_ values from 1.1 to 10) with vaccination programs achieving 70%, 80%, and 90% coverage. 

## 3. Results

### 3.1. Vaccination Coverage Required to Establish Herd Immunity against SARS-CoV-2

**Figure 1:**
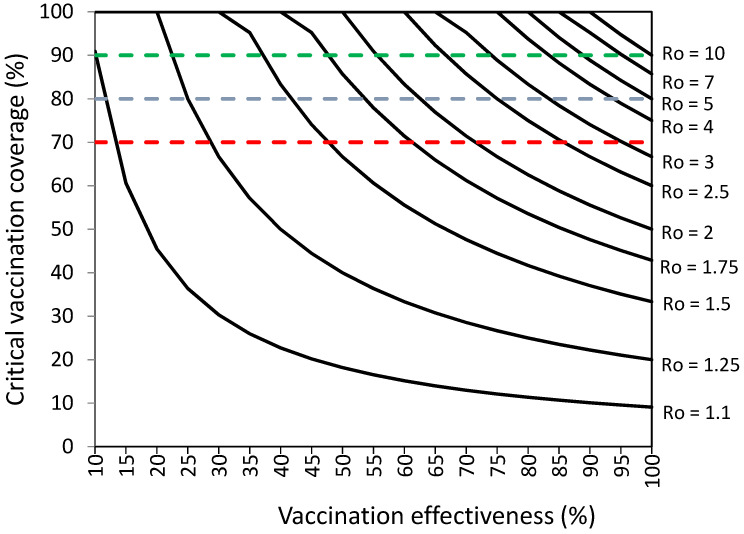
Vaccination coverage (%) required to establish herd immunity against SARS-CoV-2 with different reproductive numbers (Ro) by vaccination effectiveness (%). Objectives of vaccination coverage of 70%, 80%, and 90% are indicated with dashed red, blue, and green lines, respectively.

Figure 1 and Supplementary Materials Table S2 present the vaccination coverage required to establish herd immunity against SARS-CoV-2 without infections among vaccinated individuals. 

Figure 1 and Supplementary Materials Table S2 suggest that COVID-19 vaccination programs should achieve high percentages of vaccination coverage and high levels of COVID-19 vaccination effectiveness in order to establish herd immunity against SARS-CoV-2 with high levels of transmissibility (R_o_ values). COVID-19 vaccination programs achieving 70% vaccination effectiveness could establish herd immunity against viruses with R_o_ ranging from 1.1 to 3.25 with percentages of vaccination coverage ranging from 13% to 98.9%, respectively. Vaccination programs achieving 80–100% vaccination effectiveness could establish herd immunity against viruses with R_o_ ranging from 1.1 to 10 with percentages of vaccination coverage ranging from 9–10% to 90–100%, respectively. 

### 3.2. Vaccination Coverage Required to Establish Herd Immunity against SARS-CoV-2 with Infections among Vaccinated Individuals

**Figure 2:**
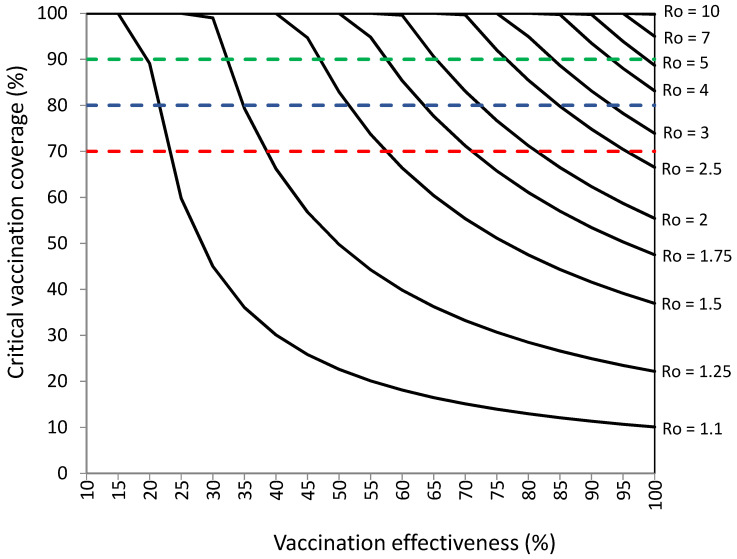
Vaccination coverage (%) required to establish herd immunity against SARS-CoV-2 with different reproductive numbers (R_o_) by vaccination effectiveness (%) in a population with 9.8% of infections among vaccinated individuals. Objectives of vaccination coverage of 70%, 80%, and 90% are indicated with dashed red, blue, and green lines, respectively.

Figure 2 and Table 2 show that COVID-19 vaccination programs should achieve higher percentages of vaccination coverage and higher levels of effectiveness to establish herd immunity than those required without infections among vaccinated individuals. COVID-19 vaccination could establish herd immunity against SARS-CoV-2 with R_o_ values ranging from 1.1 to 10, percentages of vaccination coverage of 10–100%, and levels of vaccination effectiveness of 10–100%, when 9.8% of infections occur among vaccinated individuals. The required percentages of vaccination coverage would be 74–100% against viruses with R_o_ values of 3–10, but vaccination effectiveness should be 80–100%.

The critical percentages of vaccination coverage were 1–96% higher than those without infections among vaccinated individuals (presented in Figure 1), with higher vaccination coverage increases for lower levels of vaccination effectiveness. The required percentages of vaccination coverage were 1–24% higher than those without infections among vaccinated individuals for levels of vaccination effectiveness ≥ 50% and 20–96% higher than those without infections among vaccinated individuals for levels of vaccination effectiveness < 50%.

Vaccination programs achieving 70% vaccination effectiveness could establish herd immunity against viruses with R_o_ ranging from 1.1 to 2.25 with percentages of vaccination coverage ranging from 15.1% to 92.3%, respectively. Vaccination programs achieving 80% vaccination effectiveness could establish herd immunity against viruses with R_o_ ranging from 1.1 to 3 with percentages of vaccination coverage ranging from 13% to 98.6%. Vaccination programs achieving 90% vaccination effectiveness could establish herd immunity against viruses with R_o_ ranging from 1.1 to 4.75 with percentages of vaccination coverage ranging from 11.3% to 99.8%. Vaccination programs achieving 100% vaccination effectiveness could establish herd immunity against viruses with R_o_ ranging from 1.1 to 10 with percentages of vaccination coverage ranging from 10.1% to 99.8%. 

In close settings, very high percentages of vaccination coverage are required to block SARS-CoV-2 transmission due to their 75% higher transmissibility. For example, the vaccination coverage is 89.1% against viruses with R_o_ = 2 (R_o_ = 3.5 in the close setting) and 90% vaccination effectiveness (Figure 2, Table 2). In places with high-risk individuals, very high levels of vaccination coverage are required to block SARS-CoV-2 transmission due to their lower levels of vaccine effectiveness. For example, the vaccination coverage is 92.3% against viruses with R_o_ = 2.25 and 90% vaccination effectiveness (72% in high-risk individuals) (Figure 2, Table 2). 

**Figure 3:**
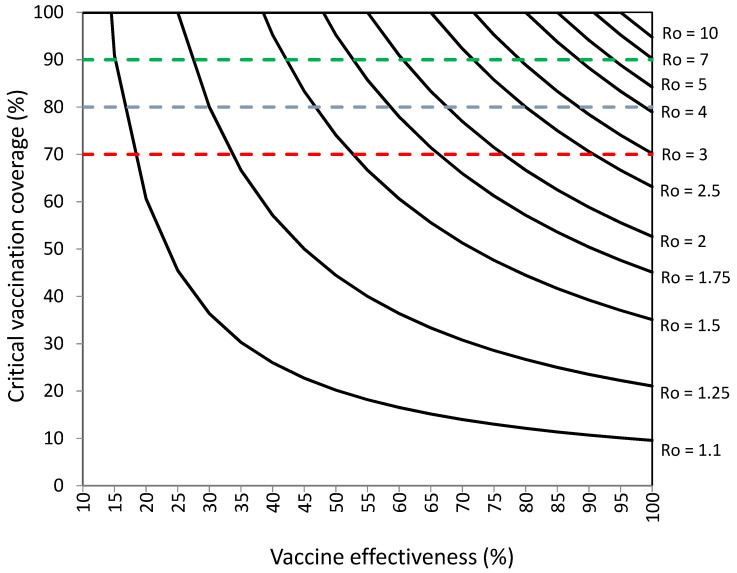
Vaccination coverage (%) required to establish herd immunity against SARS-CoV-2 with different reproductive numbers (R_o_) by vaccination effectiveness (%) in a population with 5% of infections among vaccinated individuals. Objectives of vaccination coverage of 70%, 80%, and 90% are indicated with dashed red, blue, and green lines, respectively.

Table 3 and Figure 3 present the vaccination coverage that would be required to establish herd immunity against SARS-CoV-2 with 5% of infections among vaccinated individuals for different levels of vaccination effectiveness.

Table 3 and Figure 3 show that critical percentages of vaccination coverage would be 1–11% higher than those without infections among vaccinated individuals (presented in Figure 1 and Supplementary Materials Table S2) for levels of vaccination effectiveness ≥ 50% and 14–33% higher for levels of vaccination effectiveness < 50%.

### 3.3. Assessment of Herd Immunity Levels Achieved with Percentages of Vaccination Coverage of 70%, 80%, and 90%

Table 4 presents the levels of COVID-19 vaccination effectiveness necessary to establish herd immunity against SARS-CoV-2 with R_o_ values from 1.1 to 10 when vaccination coverage is 70%, 80%, and 90%, with 0% and 9.8% of infections among vaccinated individuals. The results presented in this table show that infections among vaccinated individuals reduce the herd immunity effects of COVID-19 vaccination programs due to two negative effects: (1) Vaccination programs could establish herd immunity against SARS-CoV-2 with R_o_ values lower than those without infections among vaccinated individuals. (2) The vaccination effectiveness levels necessary to establish herd immunity against SARS-CoV-2 are higher than those without infections among vaccinated individuals. 

The results presented on Table 4, as well as those presented in Figure 2 and Table 2, suggest that the vaccination coverage objective of 70%, proposed by the WHO, could be sufficient to establish herd immunity against SARS-CoV-2 with R_o_ values from 1.1 to 2.5 when there are 9.8% of infections among vaccinated individuals. Alternative vaccination coverage objectives of 80% and 90% could be sufficient against viruses with R_o_ values from 1.1 to 3.5 and from 1.1 to 5, respectively. 

Without infections among vaccinated individuals, vaccination coverage objectives of 70%, 80%, and 90% could be sufficient against viruses with R_o_ values from 1.1 to 3.25, 1.1 to 5, and 1.1 to 10, respectively (Figure 1, Table S2). With 5% of infections among vaccinated individuals, vaccination coverage objectives of 70%, 80%, and 90% could be sufficient against viruses with R_o_ values from 1.1 to 2.75, 1.1 to 4, and 1.1 to 6, respectively (Figure 3 Table 3).

Table 5 and Table 6 present the R_o_ values of SARS-CoV-2 that the COVID-19 vaccines could block by establishing herd immunity with 70%, 80% and 90% vaccination coverage, when 9.8% of infections occur among vaccinated individuals and when infections do not occur among vaccinated individuals, respectively. When 9.8% infections occur among vaccinated individuals, COVID-19 vaccines could establish herd immunity against the Omicron variant with R_o_ values ranging from 1.09 to 1.51 and against the Delta variant with R_o_ values ranging from 1.56 to 3.71, with higher R_o_ values for vaccination programs achieving 90% vaccination coverage (Table 5).

When infections do not occur among vaccinated individuals, the COVID-19 vaccines could establish herd immunity against the Omicron variant with R_o_ values ranging from 1,18 to 1.76 and against the Delta variant with R_o_ values ranging from 1.75 to 5.52, with higher R_o_ values for vaccination programs achieving 90% vaccination coverage. In this situation, COVID-19 vaccination programs could establish herd immunity against SARS-CoV-2 with R_o_ values 7–13% higher than those obtained with 9.8% of infections among vaccinated individuals (Table 6). 

In individuals vaccinated with three doses of the Pfizer vaccine (PF 3D vaccination status) and in those vaccinated with two doses of the AstraZeneca vaccine plus a booster dose of an RNA vaccine (AZ 3P vaccination status), 77% and 73% effectiveness in preventing symptomatic Omicron infection and 93.8% and 94.3% effectiveness in preventing symptomatic Delta infection, respectively, have been obtained [28]. Consequently, these vaccination strategies could block the transmission of SARS-CoV-2 with higher R_o_ values than those obtained with other COVID-19 vaccination strategies presented in Table 5 and Table 6. When 9.8% infections occur among vaccinated individuals, the AZ 3D and PF 3D vaccination strategies could establish herd immunity against the Omicron variant with R_o_ values ranging from 1.79 to 2.53 and against the Delta variant with R_o_ values ranging from 2.43 to 4.26 (Table 5). 

### 3.4. Vaccination Coverage Required to Establish Herd Immunity against SARS-CoV-2 When Part of the Population Is Already Protected

**Figure 4:**
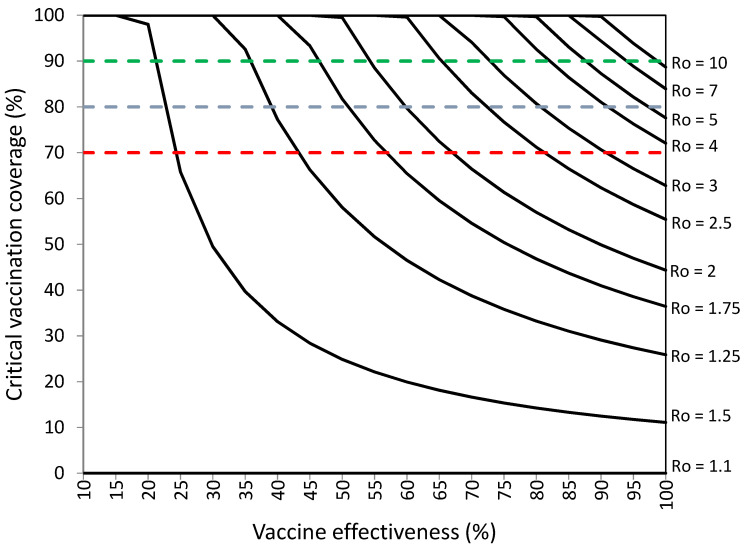
Vaccination coverage (%) required to establish herd immunity against SARS-CoV-2 with different basic reproductive numbers (R_o_) by vaccination effectiveness (%) in a population with 10% prevalence of protected individuals and 9.8% of infections among vaccinated individuals. Objectives of vaccination coverage of 70%, 80%, and 90% are indicated with dashed red, blue, and green lines, respectively.

Figure 4 and Supplementary Materials Table S3 present the percentages of vaccination coverage required to establish herd immunity against SARS-CoV-2 for levels of COVID-19 vaccination effectiveness from 10% to 100% when the prevalence of protected individuals is 10% and 9.8% of infections occur among vaccinated individuals. 

Figure 4 shows that the critical percentages of vaccination coverage are quite similar to those without 9.8% infections among vaccinated individuals (Figure 1) when 10% of the population is already protected against SARS-CoV-2 and 9.8% infections occur among vaccinated individuals. The critical vaccination coverage was 14% for viruses with R_o_ = 1.25 and vaccine effectiveness of 80%, 80% for viruses with R_o_ = 2 and vaccine effectiveness of 60%, and 90% for viruses with R_o_ = 3.75 and vaccine effectiveness of 80%.

**Figure 5:**
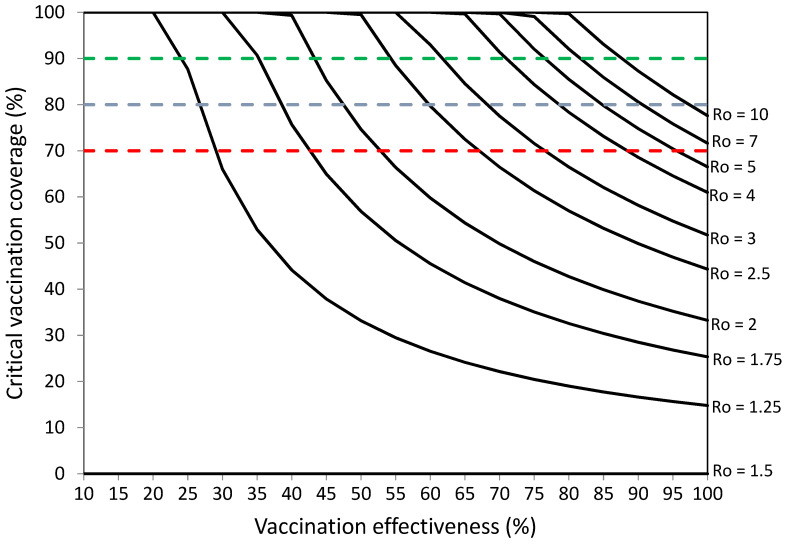
Vaccination coverage (%) required to establish herd immunity against SARS-CoV-2 with different basic reproductive numbers (R_o_) by vaccination effectiveness (%) in a population with 20% prevalence of protected individuals and 9.8% of infections among vaccinated individuals. Objectives of vaccination coverage of 70%, 80%, and 90% are indicated with dashed red, blue, and green lines, respectively.

Figure 5 and Supplementary Materials Table S4 present the percentages of vaccination coverage required to establish herd immunity against SARS-CoV-2 for levels of COVID-19 vaccination effectiveness from 10% to 100% in a population with a prevalence of protected individuals of 20% and 9.8% infections among vaccinated individuals. 

In a community with a prevalence of protected individuals of 20% (Figure 5), the vaccination coverage required is 45% for viruses with R_o_ = 1.75 and vaccine effectiveness of 80%, 60% for viruses with R_o_ = 2 and vaccine effectiveness of 60%, and 76% for viruses with R_o_ = 3.75 and vaccine effectiveness of 80%. In a community with a prevalence of protected individuals of 25%, the vaccination coverage required is 25% for viruses with R_o_ = 1.75 and vaccine effectiveness of 80%, 50% for viruses with R_o_ = 2 and vaccine effectiveness of 60%, and 96% for viruses with R_o_ = 3.75 and vaccine effectiveness of 60%.

Figure 4 and Figure 5 suggest that vaccination would not be necessary to establish herd immunity against viruses with R_o_ = 1.1 and R_o_ = 1.25 when the prevalence of protected individuals is ≥10% and ≥20%, respectively, because the critical prevalence of protected individuals necessary to establish herd immunity is already achieved without vaccination. By contrast, percentages of vaccination coverage >70% are necessary to establish herd immunity against viruses with R_o_ ≥ 2 when 10% of the population is already protected and vaccination effectiveness is lower than 70%, against viruses with R_o_ ≥ 2.25 when 20% of the population is already protected and vaccination effectiveness is lower than 70%, and against viruses with R_o_ ≥ 2.75 when 25% of the population is already protected and vaccination effectiveness is lower than 70%.

## 4. Discussion

This study assessed the percentages of COVID-19 vaccination coverage that would be required to establish herd immunity against SARS-CoV-2 in the population of countries and regions. When COVID-19 vaccines became available on 2021, it was expected that vaccination coverage of 70% would be sufficient to establish anti-SARS-CoV-2 herd immunity in the population [9,10]. Nevertheless, the results obtained in this study suggest that higher percentages of vaccination coverage and higher levels of vaccination effectiveness are necessary to block the transmission of Omicron and other emergent SARS-CoV-2 variants that have a greater infectious capacity. 

The WHO has proposed to achieve 70% COVID-19 vaccination coverage in all countries of the world by mid-2022 [9,38]. The study suggests that achieving 70% vaccination coverage could be sufficient to prevent the transmission of SARS-CoV-2 with low and moderate transmissibility (R_o_ values from 1.1 to 3.25) but not against Omicron and other emergent SARS-CoV-2 variants with greater infectious capacity. Percentages of vaccination of 70% could be sufficient to prevent serious disease, but they are not sufficient to block community transmission of Omicron and other SARS-CoV-2 variants with greater infectious capacity. 

The vaccination coverage objectives of 80% and 90% could be sufficient to prevent the transmission of SARS-CoV-2 with high transmissibility (R_o_ values from 3.25 to 5) and very high transmissibility (R_o_ values > 5), respectively. Consequently, a general vaccination coverage objective of 90% is the most effective in blocking the transmission of Omicron and other SARS-CoV-2 variants with R_o_ higher than 5. 

The findings of this study suggest that the effectiveness of COVID-19 vaccination should be increased because vaccination coverages of 80% and 90% could be sufficient to establish herd immunity against SARS-CoV-2 with R_o_ values of 3.25 to 5 and >5, respectively, only when vaccination effectiveness in preventing SARS-CoV-2 infection is >87% and >88%, respectively. The effectiveness of the available COVID-19 vaccines in preventing Omicron infection range from 22% to 44% [8]. A study carried out in 2021 by Hodgson et al. assessing the potential for COVID-19 vaccination to generate herd immunity concluded that if highly transmissible variants become dominant in areas with low seroprevalence, the control of infection by vaccination without non-pharmaceutical interventions may only be achievable with a vaccine effectiveness ≥ 80% [39].

Higher levels of vaccination coverage are required to establish herd immunity against SARS-CoV-2 in close or confined settings and in places with high-risk individuals because of high transmission levels and lower COVID-19 vaccination effectiveness, respectively. Similar results have been obtained for influenza vaccination [40]. 

Required percentages of vaccination coverage would be lower if 10–20% of the population were already protected against SARS-CoV-2 because of natural infections. Similar results have been obtained for influenza vaccination [40]. In this situation, however, COVID-19 vaccination should be recommended for all individuals for several reasons. First, COVID-19 vaccination prevents infections, especially in high-risk individuals. Second, it is necessary to conduct seroprevalence studies in representative samples of the population to estimate the prevalence of protected individuals in the population. Third, a complex and costly screening and vaccination program should be developed, in which all individuals in the population must be screened for SARS-CoV-2 antibodies and vaccines should be given only unprotected individuals. 

The findings of this study found that Omicron infections among vaccinated individuals reduced herd immunity effects of COVID-19 vaccines for three reasons: (1) the percentages of vaccination coverage required to establish herd immunity in the population were higher than those without infections among vaccinated individuals; (2) vaccination programs established herd immunity against SARS-CoV-2 with R_o_ values lower than those without infections among vaccinated individuals; and (3) levels of vaccination effectiveness necessary to establish herd immunity against SARS-CoV-2 were higher than those without infections among vaccinated individuals. 

This study suggests that vaccination programs could not establish herd immunity against Omicron and other SARS-CoV-2 with greater infectious capacity using available vaccines because of their <50% effectiveness in preventing Omicron infection [8]. However, COVID-19 vaccination programs could establish herd immunity against Omicron and other SARS-CoV-2 variants with R_o_ values higher than 5 by achieving >90% vaccination effectiveness and >90% vaccination coverage. Using a vaccination strategy using three doses of the Pfizer vaccine (PF 3D vaccination status) and two doses of the AstraZeneca vaccine plus a booster dose of an mRNA vaccine (AZ 3P vaccination status), herd immunity could not be established against Omicron and other SARS-CoV-2 variants with R_o_ values higher than 5 because their effectiveness in preventing symptomatic Omicron infection are 77% and 73%, respectively [28]. 

What should be done to establish herd immunity to block transmission of Omicron in the population? Four measures should be implemented: (1) increase the vaccination coverage to at last 90%; (2) increase the effectiveness of COVID-19 vaccines in preventing Omicron infection to 80–90%; (3) administer booster doses based on waning immunity protection data; and (4) develop universal COVID-19 vaccination programs in all countries.

The available COVID-19 vaccines are effective in preventing severe disease and mortality from different SARS-CoV-2 variants, but they are less effective in preventing Omicron infection [7,8,27,28]. The effectiveness in preventing severe Omicron infection was 71–73% using the Astra/Zeneca, Moderna, and Pfizer/BioNTech vaccines and 37–67% using other vaccines (Table 1). A network meta-analysis assessing the relative efficacy of seven vaccines (Pfizer/BioNTech, Moderna, AstraZeneca, Janssen, Gamaleya, Sinopharm, Sinovac) in preventing symptomatic and severe COVID-19 found that the probability of being better than other competing vaccines in terms of efficacy in preventing severe COVID-19 was not statistically significant [29]. By contrast, the effectiveness in preventing Omicron infection is 43–48% using the Moderna, Pfizer/BioNTech, Sputnik, and Novavax vaccines and 24–38% using other vaccines [8] (Table 1). Consequently, a main challenge to overcome is increasing the effectiveness in preventing Omicron infection to higher than 80% or 90%. 

Infections among vaccinated individuals can be explained by waning vaccine-induced immunity protection and by the emergence of SARS-CoV-2 variants escaping immune response, resulting in lower levels of COVID-19 vaccination effectiveness [33,34,35,36,37]. The contribution of each factor remains unknown. The immunity escape of Omicron is responsible for the lower levels of effectiveness in preventing Omicron infection [11,12]. The information on reinfection rates and infection rates among vaccinated individuals is important for public health decision making. First, higher percentages of vaccination coverage should be achieved to block SARS-CoV-2 transmission when infections occur among vaccinated individuals. Second, mask-wearing and other preventive behaviours could be used to reduce SARS-CoV-2 transmission from infected vaccinated individuals. 

All countries of the world should implement universal or mass COVID-19 vaccination programs. Vaccination programs focused on specific population groups, such as older and high-risk individuals, cannot generate herd immunity in the population. Stratified COVID-19 vaccination, in which population groups are vaccinated in several phases, have been implemented in most countries. In the first phase, higher priority is placed on vaccinating older and high-risk individuals to reduce morbidity and mortality from COVID-19. Other adults, adolescents, and children are then vaccinated in subsequent phases. The objective of this strategy is to reduce the morbidity and mortality from COVID-19. Nevertheless, herd immunity against Omicron and other SARS-CoV-2 variants can be established only by achieving high percentages of vaccination coverage in all population groups. From a practical point of view, herd immunity can be considered established in a population group when the vaccination coverage is equal to or higher than the critical coverage [41]. Nevertheless, herd immunity could be considered established in an entire population when the vaccination coverage is equal to or higher than the critical coverage in all population groups. 

The study has several limitations. First, percentages of vaccination coverage that would be required to establish herd immunity against SARS-CoV-2 were determined taking into account SARS-CoV-2 transmissibility and COVID-19 vaccination effectiveness. This method is based on the following assumptions: (1) homogeneous mixing of individuals within the population and (2) homogeneous distribution of protected individuals within the population [17,18,19,20,21]. If the distribution of protected individuals is not homogeneous, required percentages of vaccination coverage would be higher or lower in places with lower or higher levels of protected individuals, respectively. Nevertheless, it is possible to assume homogeneous mixing of persons and a homogeneous distribution of protected individuals within vaccinated population groups [20,21]. Second, percentages of vaccination coverage that would be required to establish herd immunity were determined for SARS-CoV-2 with values of R_o_ from 1.1 to 10. Required percentages of vaccination coverage could be lower using measures for preventing SARS-CoV-2 transmission, such as mask-wearing and physical distancing. Nevertheless, the levels of COVID-19 vaccination effectiveness are higher than those achieved with mask-wearing and social distancing [42,43]. Third, percentages of vaccination coverage that would be required to establish herd immunity were determined for SARS-CoV-2 with R_o_ values from 1.1 to 10. Higher critical percentages of vaccination would be required against new SARS-CoV-2 variants with R_o_ values higher than 10. Nevertheless, the range of R_o_ values assumed in this study has been found in studies assessing the R_o_ values of SARS-CoV-2 [20,21,22,23,24], and the transmissibility of future variants should be in the range of values assumed in the study. Fourth, the study determined the percentages of vaccination coverage that would be required to establish herd immunity with 0%, 5%, and 9.8% infection rates among vaccinated individuals. Higher critical percentages of vaccination would be required against SARS-CoV-2 variants with infections rates higher than 9.8%. Nevertheless, it is possible to assume infection rates of 0–9.8% for new SARS-CoV-2 variants because an infection rate of 9.8% has been observed for Omicron [11], and future vaccines could be associated with a higher efficacy and effectiveness in preventing infections among vaccinated individuals. 

COVID-19 vaccination is the key preventive measures included in the COVID-19 Strategic Preparedness and Response Plan (SPRP) of the WHO [43]. The objectives of the SPRP are to suppress transmission, reduce exposure, prevent infection, and reduce morbidity and mortality from COVID-19 [43]. COVID-19 vaccination must be deployed in combination with other preventive measures and diagnostic and therapeutic activities to constitute a strong and effective response to the COVID-19 pandemic. 

The strategy proposed by the WHO to achieve high percentages of vaccination coverage (at least 70% by mid-2022) considers three phases: (1) vaccination of older adults and high-risk groups of all ages; (2) extensive vaccination of the full adult age group; and (3) extensive vaccination of adolescents [44]. The objective of the first vaccination step is to reduce morbidity and mortality from COVID-19, while the objectives in the second and third vaccination steps are also to reduce SARS-CoV-2 transmission [44]. 

Each country should use vaccination as a tool to reach a “new normal” in which social and economic activity are resumed to the greatest extent possible while minimizing negative impacts [44]. The WHO proposes four levels of COVID-19 vaccination coverage: low, medium, high, and very high [44]. Low and medium levels of vaccination coverage must be complemented with intensive prevention measures, because movements along the socioeconomic dimension increase SARS-CoV-2 transmission (i.e., basic reproductive numbers). High and very high levels of vaccination coverage must be achieved in countries that are moving toward normal social and economic activity, because, in this situation, their prevention measures could be reduced. The results obtained in this study suggest that, in countries moving toward normal social and economic activity, vaccination coverage objectives should be determined by taking into account the critical percentages of vaccination coverage required to block the SARS-CoV-2 transmission in the community, based on the transmissibility of SARS-CoV-2 (R_o_ values) and vaccination effectiveness. 

As of 10 April 2022, the rates of complete COVID-19 vaccination were as follows: 58.2% worldwide, 73.2% in South America, 67.7% in Asia, 65.3% in Europe, 62.9% in Oceania, 62.7% in North America, and 15.4% in Africa [4]. These percentages indicate that COVID-19 vaccination coverage should be increased in most countries to achieve the objective of blocking the transmission of SARS-CoV-2 throughout the world.

Several challenges must be overcome in creating herd immunity against SARS-CoV-2 by mass vaccination in different countries of the world [10]. First, advanced vaccination programs should be developed to achieve percentages of vaccination coverage higher than 70–90%. Second, the vaccines and resources required to achieve herd immunity should be assessed [38,44]. Third, vaccination access must be increased in countries and regions with low percentages of COVID-19 vaccination coverage [44,45,46,47]. Fourth, effective communication strategies should be developed to reduce vaccination hesitance [48,49] and increase vaccination confidence and acceptance [50,51,52]. Health education information should show that COVID-19 vaccines provide individual protection in vaccinated individuals and that they protect vulnerable and unprotected individuals by reducing the transmission of SARS-CoV-2 in the population. Nevertheless, it is also necessary to use vaccines that are at least 80–90% effective in preventing Omicron infections.

The European Medicines Agency (EMA) approves the marketing authorization of COVID-19 vaccines based on their safety and efficacy demonstrated in adults, high-risk individuals, and individuals aged 65 years or older based on the results obtained in at least one well-designed large-scale phase 3 efficacy trial [53]. The EMA has indicated that the primary endpoint in pivotal vaccine efficacy trials should be laboratory-confirmed COVID-19 disease of any severity, and the secondary endpoints should include estimates of protection against symptomatic and severe disease [53]. The endpoints of phase 3 trials should include estimates of effectiveness in preventing SARS-CoV-2 infection to assess whether COVID-19 vaccines are effective in blocking SARS-CoV-2 transmission. 

## 5. Conclusions

High percentages of vaccination coverage and high levels of vaccination effectiveness are necessary to block the transmission of Omicron and other SARS-CoV-2 variants with greater infectious capacity. 

Two measures should be implemented to establish herd immunity against SARS-CoV-2: (1) achieve ≥ 90% COVID-19 vaccination coverage worldwide, and (2) increase the effectiveness of COVID-19 vaccines in preventing Omicron infection to at least 88%. 

## Figures and Tables

**Table 1:** Effectiveness of the available COVID-19 vaccines. Data from the Institute for Health Metrics and Evaluation (IHME) [8].

Vaccine	Effectiveness (%) in Preventing					
	Ancetral Variant		Delta Variant		Omicron Variant	
	Severe Disease	Infection	Severe Disease	Infection	Severe Disease	Infection
Moderna	97	92	97	91	73	48
Pfizer/BioNTech	95	86	95	84	72	44
Sputnik	92	86	89	85	67	44
Novavax	89	83	86	82	65	43
Covaxin	78	73	76	72	57	38
Oxford/Astra-Zeneca	94	63	94	69	71	36
Sinopharm	73	68	71	67	53	35
Janssen	86	72	76	64	57	33
CoronaVac	50	47	49	46	37	24
Convidecia	66	62	64	61	48	22

**Table 2:** Critical vaccination coverage (V_c_) required to establish herd immunity against SARS-CoV-2 with reproductive numbers (R_o_) from 1.1 to 10 by vaccination effectiveness (%), with 9.8% of infections among vaccinated individuals.

Ro of SARS-CoV-2 ^b^	Critical Vaccination Coverage (%) ^a^ For COVID-19 Vaccination Effectiveness from 10% to 100%									
	10%	20%	30%	40%	50%	60%	70%	80%	90%	100%
1.1	−	89.1	45.0	30.1	22.6	18.1	15.1	13.0	11.3	10.1
1.25	−	−	99.0	66.2	49.8	39.8	33.2	28.5	24.9	22.2
1.5	−	−	−	−	82.9	66.4	55.4	47.5	41.6	37.0
1.75	−	−	−	−	−	85.4	71.2	61.1	53.4	47.5
2	−	−	−	−	−	99.6	83.1	71.2	62.3	55.4
2.25	−	−	−	−	−	−	92.3	79.1	69.3	61.6
2.5	−	−	−	−	−	−	99.7	85.5	74.8	66.5
2.75	−	−	−	−	−	−	−	90.7	79.3	70.6
3	−	−	−	−	−	−	−	95.0	83.1	73.9
3.25	−	−	−	−	−	−	−	98.6	86.3	76.8
3.5	−	−	−	−	−	−	−	−	89.1	79.2
3.75	−	−	−	−	−	−	−	−	91.4	81.3
4	−	−	−	−	−	−	−	−	93.5	83.1
4.25	−	−	−	−	−	−	−	−	95.3	84.8
4.5	−	−	−	−	−	−	−	−	97.0	86.2
4.75	−	−	−	−	−	−	−	−	98.4	87.5
5	−	−	−	−	−	−	−	−	99.8	88.7
6	−	−	−	−	−	−	−	−	−	92.4
7	−	−	−	−	−	−	−	−	−	93.8
8	−	−	−	−	−	−	−	−	−	98.5
9	−	−	−	−	−	−	−	−	−	97.0
10	−	−	−	−	−	−	−	−	−	99.8

^a^ V_c_ = I_c_/Effectiveness. I_c_ = 1 − (1/R_o_). The critical vaccination coverage is not indicated (−) when herd immunity could not be established with 100% vaccination coverage. ^b^ The basic reproduction number R_o_ indicates the average number of secondary cases generated per infected case in a completely susceptible population.

**Table 3:** Critical vaccination coverage (V_c_) required to establish herd immunity against SARS-CoV-2 with reproductive numbers (R_o_) from 1.1 to 10 by vaccination effectiveness (%), with 5% of infections among vaccinated individuals.

R_o_ of SARS-CoV-2 ^b^	Critical Vaccination Coverage (%) ^a^ for COVID-19 Vaccination Effectiveness from 10% to 100%									
	10%	20%	30%	40%	50%	60%	70%	80%	90%	100%
1.1	−	60.6	36.4	26.0	20.2	16.5	14.0	12.1	10.7	9.6
1.25	−	−	80.0	57.1	44.4	36.4	30.8	26.7	23.5	21.1
1.5	−	−	−	95.2	74.1	60.6	51.3	44.4	39.2	35.1
1.75	−	−	−	−	95.2	77.9	65.9	57.1	50.4	45.1
2	−	−	−	−	−	90.9	76.9	66.7	58.8	52.6
2.25	−	−	−	−	−	−	85.5	74.1	65.4	58.5
2.5	−	−	−	−	−	−	92.3	80.0	70.6	63.2
2.75	−	−	−	−	−	−	97.9	84.8	74.9	67.0
3	−	−	−	−	−	−	−	88.9	78.4	70.2
3.25	−	−	−	−	−	−	−	92.3	81.4	72.9
3.5	−	−	−	−	−	−	−	95.2	84.0	75.2
3.75	−	−	−	−	−	−	−	97.8	86.3	77.2
4	−	−	−	−	−	−	−	100	88.2	78.9
4.25	−	−	−	−	−	−	−	−	90.0	80.5
4.5	−	−	−	−	−	−	−	−	91.5	81.9
4.75	−	−	−	−	−	−	−	−	92.9	83.1
5	−	−	−	−	−	−	−	−	94.1	84.2
6	−	−	−	−	−	−	−	−	98,0	87.7
7	−	−	−	−	−	−	−	−	−	90.2
8	−	−	−	−	−	−	−	−	−	92.1
9	−	−	−	−	−	−	−	−	−	93.6
10	−	−	−	−	−	−	−	−	−	94.7

^a^ V_c_ = I_c_/Effectiveness. I_c_ = 1 − (1/R_o_). The critical vaccination coverage is not indicated (−) when herd immunity could not be established with 100% vaccination coverage; ^b^ The basic reproduction number R_o_ indicates the average number of secondary cases generated per infected case in a completely susceptible population.

**Table 4:** Vaccination effectiveness necessary to establish herd immunity against SARS-CoV-2 with the basic reproduction number ranging from 1.1 to 10 in populations with percentages of vaccination coverage of 70%, 80%, and 90%, with 0% and 9.8% of infections among vaccinated individuals.

R_o_ of SARS-CoV-2	COVID-19 Vaccination Effectiveness (%) Required to Establish Herd Immunity with 70%, 80%, and 90% Vaccination Coverage					
	Vaccination Coverage ^a^			Vaccination Coverage and 9.8% of Infections in Vaccinated Individuals ^b^		
	70%	80%	90%	70%	80%	90%
1.1	13.0	11.4	10.1	22.8	21.2	19.9
1.25	28.6	25.0	22.2	38.4	34.8	32.0
1.5	47.6	41.7	37.0	57.4	51.5	46.8
1.75	61.2	53.6	47.6	71.0	63.4	57.4
2	71.4	62.5	55.6	81.2	72.3	65.4
2.25	79.4	69.4	61.7	89.2	79.2	71.5
2.5	85.7	75.0	66.7	95.5	84.8	76.5
2.75	90.9	79.5	70.7	−	89.3	80.5
3	95.2	83.3	74.1	−	93.1	83.9
3.25	98.9	86.5	76.9	−	96.3	86.7
3.5	−	89.3	79.4	−	99.1	89.2
3.75	−	91.7	81.5	−	−	91.3
4	−	93.8	83.3	−	−	93.1
4.25	−	95.6	85.0	−	−	94.8
4.5	−	97.2	86.4	−	−	96.2
4.75	−	98.7	87.7	−	−	97.5
5	−	100.0	88.9	−	−	98.7
6	−	−	92.6	−	−	−
7	−	−	95.2	−	−	−
8	−	−	97,2	−	−	−
9	−	−	98,8	−	−	−
10	−	−	100.0	−	−	−

^a^ Effectiveness = I_c_/Vaccination coverage. The effectiveness is not indicated (−) when herd immunity could not be established with 100% vaccination coverage; ^b^ Effectiveness = (I_c_/Vaccination coverage) + infection rate among vaccinated individuals. The effectiveness is not indicated (−) when herd immunity could not be established with 100% vaccination coverage.

**Table 5:** R_o_ of SARS-CoV-2 that COVID-19 vaccination programs could block by achieving 70%, 80%, and 90% vaccination coverage, when 9.8% of infections occur among vaccinated individuals.

Vaccine	R_o_ of SARS-CoV-2 Blocked by COVID-19 Vaccination Programs Achieving 70%, 80% and 90% Vaccination Coverage ^a^								
	Vaccination Coverage			Vaccination Coverage			Vaccination Coverage		
	70%	80%	90%	70%	80%	90%	70%	80%	90%
	Ancestral Variant			Delta Variant			Omicron Variant		
	R_o_	R_o_	R_o_	R_o_	R_o_	R_o_	R_o_	R_o_	R_o_
Moderna	2.36	2.92	3.84	2.32	2.85	3.71	1.37	1.44	1.52
Pfizer/BioNTech	2.14	2.56	3.18	2.08	2.46	3.01	1.31	1.38	1.44
Sputnik	2.14	2.56	3.18	2.11	2.51	3.09	1.31	1.38	1.44
Novavax	2.05	2.41	2.93	2.02	2.37	2.86	1.30	1.36	1.43
Covaxin	1.79	2.02	2.32	1.77	1.99	2.27	1.25	1.29	1.34
Oxford/Astra-Zeneca	1.59	1.74	1.92	1.71	1.90	2.14	1.22	1.27	1.31
Sinopharm	1.69	1.87	2.10	1.67	1.84	2.06	1.21	1.25	1.29
Janssen	1.77	1.99	2.27	1.61	1.77	1.95	1.19	1.23	1.26
CoronaVac	1.35	1.42	1.50	1.34	1.41	1.48	1.11	1.13	1.15
Convidecia	1.58	1.72	1.89	1.56	1.69	1.85	1.09	1.11	1.12
AstraZeneca (AZ 3D)				2.43	3.05	4.10	1.79	2.02	2.32
Pfizer (PF 3D)				2.47	3.13	4.26	1.89	2.16	2.53

^a^ R_o_ values obtained for levels of effectiveness in preventing SARS-CoV-2 infection for all vaccines [8] (Table 1), and effectiveness in preventing symptomatic infection for AZ 3D and PF 3D vaccination [28].

**Table 6:** R_o_ of SARS-CoV-2 that COVID-19 vaccination programs could block by achieving 70%, 80%, and 90% vaccination coverage, without infections among vaccinated individuals.

Vaccine	R_o_ of SARS-CoV-2 Blocked by COVID-19 Vaccination Programs Achieving 70%, 80%, and 90% Vaccination Coverage ^a^								
	Vaccination Coverage			Vaccination Coverage			Vaccination Coverage		
	70%	80%	90%	70%	80%	90%	70%	80%	90%
	Ancestral Variant			Delta Variant			Omicron Variant		
	R_o_	R_o_	R_o_	R_o_	R_o_	R_o_	R_o_	R_o_	R_o_
Moderna	2.81	3.79	5.81	2.75	3.68	5.52	1.51	1.62	1.76
Pfizer/BioNTech	2.51	3.21	4.42	2.43	3.05	4.10	1.45	1.54	1.66
Sputnik	2.51	3.21	4.42	2.47	3.13	4.26	1.45	1.54	1.66
Novavax	2.39	2.98	3.95	2.35	2.91	3.82	1.43	1.52	1.63
Covaxin	2.04	2.40	2.92	2.02	2.36	2.84	1.36	1.44	1.52
Oxford/Astra-Zeneca	1.79	2.02	2.31	1.93	2.23	2.64	1.34	1.40	1.48
Sinopharm	1.91	2.19	2.58	1.88	2.16	2.52	1.32	1.39	1.46
Janssen	2.02	2.36	2.84	1.81	2.05	2.36	1.30	1.36	1.42
CoronaVac	1.49	1.60	1.73	1.47	1.58	1.71	1.20	1.24	1.28
Convidecia	1.77	1.98	2.26	1.75	1.95	2.22	1.18	1.21	1.25
AstraZeneca (AZ 3D)				2.91	4.01	6.42	2.04	2.40	2.92
Pfizer (PF 3D)				2.97	4.14	6.81	2.17	2.60	3.26

^a^ R_o_ values obtained for levels of effectiveness in preventing SARS-CoV-2 infection for all vaccines [8] (Table 1), and effectiveness in preventing symptomatic infection for AZ 3D and PF 3D vaccination [28].

## Supplementary Materials

The following are available online at https://www.mdpi.com/article/10.3390/vaccines10050736/s1, Table S1: Completed COVID-19 vaccination coverage in countries of the world (April 2022). Table S2: Critical vaccination coverage (V_c_) required to establish herd immunity against SARS-CoV-2 with reproductive numbers (R_o_) from 1.1 to 10 by vaccination effectiveness. Table S3: Critical vaccination coverage (V_c_) required to establish herd immunity against SARS-CoV-2 with different basic reproductive numbers (R_o_) by COVID-19 vaccination effectiveness, with 10% of individuals protected and 9.8% of infections among vaccinated individuals. Table S4: Critical vaccination coverage (V_c_) required to establish herd immunity against SARS-CoV-2 with different basic reproductive numbers (R_o_) by vaccine effectiveness, with 20% of individuals protected and 9.8% of infections among vaccinated individuals.

## References

[1] WHO (2020). WHO Director-General’s Opening Remarks at the Media Briefing on COVID-19. https://www.who.int/director-general/speeches/detail/who-director-general-s-opening-remarks-at-the-media-briefing-on-COVID-19---11-March-2020..

[2] Zhu N., Zhang D., Wang W., Li X., Yang B., Song J., Zhao X., Huang B., Shi W., Lu R. (2020). A Novel Coronavirus from Patients with Pneumonia in China, 2019. N. Engl. J. Med..

[3] WHO WHO Coronavirus (COVID-19) Dashboard. https://covid19.who.int.

[4] Our World in Data Coronavirus (COVID-19) Vaccinations. https://ourworldindata.org/covid-vaccinations..

[5] WHO Status of COVID-19 Vaccines within WHO EUL/PQ Evaluation Process. https://extranet.who.int/pqweb/sites/default/files/documents/Status_COVID_VAX_18February2022.pdf.

[6] WHO (2020). COVID-19 Vaccine Tracker and Landscape. Geneva: World Health Organization. https://www.who.int/publications/m/item/draft-landscape-of-covid-19-candidate-vaccines.

[7] Rotshild V., Hirsh-Raccah B., Miskin I., Muszkat M., Matok I. (2021). Comparing the clinical efficacy of COVID-19 vaccines: A systematic review and network meta-analysis. Sci. Rep..

[8] The Institute for Health Metrics and Evaluation (IHME) (2022). COVID-19 Vaccine Efficacy Summary. https://www.healthdata.org/covid/COVID-19-vaccine-efficacy-summary.

[9] WHO (2021). Achieving 70% COVID-19 Immunization Coverage by Mid-2022. Statement of the Independent Allocation of Vaccines Group (IAVG) of COVAX. https://www.who.int/news/item/23-12-2021-achieving-70-COVID-19-immunization-coverage-by-mid-2022.

[10] Anderson R.M., Vegvari C., Truscott J., Collyer B.S. (2020). Challenges in creating herd immunity to SARS-CoV-2 infection by mass vaccination. Lancet.

[11] United Kingdom Security Agency (2021). SARS-CoV-2 Variants of Concern and Variants under Investigation in England. UK Security Agency: Technical Briefing 33. https://assets.publishing.service.gov.uk/government/uploads/system/uploads/attachment_data/file/1043807/technical-briefing-33.pdf.

[12] Collie S., Moultrie H., Bekker L.-G., Gray G. (2022). Effectiveness of BNT162b2 Vaccine against Omicron Variant in South Africa. N. Engl. J. Med..

[13] Goldberg L., Haas E.J., Milo R., Alroy-Preis S., Ash N., Huppert A. (2021). Waning Immunity after the BNT162b2 Vaccine in Israel. N. Engl. J. Med..

[14] Dolgin E. (2021). COVID vaccine immunity is wanning—How much does it matter?. Nature.

[15] Velavan T.P., Pollard A.J., Kremsner P.C. (2020). Herd immunity and vaccination of children for COVID-19. Int. J. Infect. Dis..

[16] Committee on Infectious Diseases (2022). COVID-19 Vaccines in Children and Adolescents. Pediatrics.

[17] Anderson R.M., May R.M. (1995). Infectious Diseases in Humans. Dynamics and Control.

[18] Fine P.E.M. (1993). Herd Immunity: History, Theory, Practice. Epidemiol. Rev..

[19] Gay N.J. (2003). The Theory of Measles Elimination: Implications for the Design of Elimination Strategies. J. Infect. Dis..

[20] Plans-Rubió P. (2012). Evaluation of the Establishment of Herd Immunity in the Population by Means of Serological Surveys and Vaccination Coverage. Hum. Vaccines Immunother..

[21] Plans-Rubió P. (2021). Vaccination Coverage for Routine Vaccines and Herd Immunity Levels against Measles and Pertussis in the World in 2019. Vaccines.

[22] Rahman B., Sadraddin E., Porreca A. (2020). The basic reproduction number of SARS-CoV-2 in Wuhan is about to die out, how about the rest of the World?. Rev. Med. Virol..

[23] Liu Y., Gayle A.A., Wilder-Smith A., Rocklöv J. (2020). The reproductive number of COVID-19 is higher compared to SARS coronavirus. J. Travel Med..

[24] Nishiura H., Ito K., Anzai A., Kobayashi T., Piantham C., Rodríguez-Morales A.J. (2022). Relative Reproduction Number of SARS-CoV-2 Omicron (B.1.1.529) Compared with Delta Variant in South Africa. J. Clin. Med..

[25] Lin Y., Ocklöv J. (2021). The reproductive number of the Delta variant of SARS-CoV-2 is far higher compared to the ancestral SARS-CoV-2 virus. J. Travel Med..

[26] Ito K., Piantham C., Nishiura H. (2021). Relative instantaneous reproduction number of Omicron SARS-CoV-2 variant with respect to the Delta variant in Denmark. J. Med. Virol..

[27] Lopez Bernal J., Andrews N., Gower C., Gallagher E., Simmons R., Thelwall S., Stowe J., Tessier E., Groves N., Dabrera G. (2021). Effectiveness of COVID-19 Vaccines against the B.1.617.2 (Delta) Variant. N. Engl. J. Med..

[28] Ferguson F., Ghani A., Cori A., Hogan A., Hinsley W., Volz E. (2021). Growth, Population Distribution and Immune Escape of the Omicron in England. Imperial College London. https://www.imperial.ac.uk/media/imperial-college/medicine/mrc-gida/2021-12-16-COVID19-Report-49.pdf.

[29] Cheng H., Peng Z., Luo W., Si S., Mo M., Zhou H., Xin X., Liu H., Yu Y. (2021). Efficacy and Safety of COVID-19 Vaccines in Phase III Trials: A Meta-Analysis. Vaccines.

[30] World Health Organization (WHO) (2009). Transmission dynamics and impact of pandemic influenza A (H1N1) 2009 virus. Wkly. Epidemiol. Rec..

[31] Centers for Disease Control and Prevention (CDC) (2010). Prevention and Control of Influenza with Vaccines. Recommendations of the Advisory Committee on Immunization Practices (ACIP). Morb. Mortal. Wkly. Rep..

[32] Embi P.J., Levy M.E., Naleway A.L., Patel P., Gaglani M., Natarajan K., Dascomb K., Ong T.C., Klein N.P., Liao I.-C. (2021). Effectiveness of 2-Dose Vaccination with mRNA COVID-19 Vaccines Against COVID-19–Associated Hospitalizations Among Immunocompromised Adults—Nine States, January–September 2021. MMWR. Morb. Mortal. Wkly. Rep..

[33] Murchu E.O., Byrne P., Carty P.G., De Gascun C., Keogan M., O’Neill M., Harrington P., Ryan M. (2022). Quantifying the risk of SARS-CoV-2 reinfection over time. Rev. Med. Virol..

[34] Hall V.J., Foulkes S., Charlett A., Atti A., Monk E.J.M., Simmons R., Wellington E., Cole M.J., Saei A., Oguti B. (2021). SARS-CoV-2 infection rates of antibody-positive compared with antibody-negative health-care workers in England: A large, multicentre, prospective cohort study (SIREN). Lancet.

[35] Pulliam J.R.C., Schalkwyk C., Govender N., Gottberg A., Cohen C., Groome M.J. (2021). Increased Risk of SARS-CoV-2 Reinfection Associated with Emergence of the Omicron Variant in South Africa. medRxiv.

[36] Townsend J.P., Hassler H.B., Wang Z., Miura S., Singh J., Kumar S., Ruddle N.H., Galvani A.P., Dornburg A. (2021). The durability of immunity against reinfection by SARS-CoV-2: A comparative evolutionary study. Lancet Microbe.

[37] Sui Y., Bekele Y., Berzofsky J.A. (2021). Potential SARS-CoV-2 Immune Correlates of Protection in Infection and Vaccine Immunization. Pathogens.

[38] WHO Strategy to Achieve Global COVID-19 Vaccination by Mid 2022. https://reliefweb.int/report/world/strategy-achieve-global-covid-19-vaccination-mid-2022.

[39] Hodgson D., Flasche S., Jit M., Kucharski A.J., CMMID COVID-19 Working Group (2021). The potential for vaccination-induced herd immunity against the SARS-CoV-2 B.1.1.7 variant. Euro Surveill..

[40] Plans-Rubió P. (2012). The Vaccination Coverage Required to Establish Herd Immunity against Influenza Viruses. Prev. Med..

[41] Plans P. (2013). New preventive strategy to eliminate measles, mumps and rubella from Europe based on the serological assessment of herd immunity levels is the population. Eur. J. Clin. Microbiol. Infect. Dis..

[42] Chenga V.C., Wong S., Chuangc V.W., Soa S.Y., Chena J.H., Sridhar S., To K.K., Chand J.F., Hunge V.F., Ho P. (2020). The role of community-wide wearing of face mask for control of coronavirus disease 2019 (COVID-19) epidemic due to SARS-CoV-2. J. Infect..

[43] WHO (2021). COVID-19 Strategic Preparedness and Response Plan (SPRP). Geneva: WHO. https://www.who.int/publications/i/item/WHO-WHE-2021.02.

[44] WHO COVID-19 Vaccination—Strategic Vision for 2022. https://cdn.who.int/media/docs/default-source/immunization/sage/covid/global-COVID-19-vaccination-strategic-vision-for-2022_sage-yellow-book.pdf?sfvrsn=4827ec0d_5.

[45] Anastasiou O.E., Heger D. (2021). Understanding the Influence of Individual and Systemic Factors on Vaccination Take-Up in European Citizens Aged 55 or Older. Vaccines.

[46] Rhoda D.A., Prier M.L., Clary C.B., Trimner M.K., Velandia-Gonzalez M., Danovaro-Holliday M.C., Cutts F.T. (2021). Using Household Surveys to Assess Missed Opportunities for Simultaneous Vaccination: Longitudinal Examples from Colombia and Nigeria. Vaccines.

[47] Hogan A.B., Winskill P., Watson O.J., Walker P.G.T., Whittaker C., Baguelin M., Haw D., Løchen A., Gaythorpe K.A.M., Imperial College COVID-19 Response Team (2020). Modelling the Allocation and Impact of a COVID-19 Vaccine. Imperial College London. https://www.imperial.ac.uk/media/imperial-college/medicine/mrc-gida/2020-09-25-COVID19-Report-33.pdf.

[48] Spinewine A., Pétein C., Evrard P., Vastrade C., Laurent C., Delaere B., Henrard S. (2021). Attitudes towards COVID-19 Vaccination among Hospital Staff—Understanding What Matters to Hesitant People. Vaccines.

[49] Okubo R., Yoshioka T., Ohfuji S., Matsuo T., Tabuchi T. (2021). COVID-19 Vaccine Hesitancy and Its Associated Factors in Japan. Vaccines.

[50] Popa G.L., Muntean A.-A., Muntean M.-M., Popa M.I. (2020). Knowledge and Attitudes on Vaccination in Southern Romanians: A Cross-Sectional Questionnaire. Vaccines.

[51] Kerekes S., Ji M., Shih S.-F., Chang H.-Y., Harapan H., Rajamoorthy Y., Singh A., Kanwar S., Wagner A.L. (2021). Differential Effect of Vaccine Effectiveness and Safety on COVID-19 Vaccine Acceptance across Socioeconomic Groups in an International Sample. Vaccines.

[52] Almalki M.J., Alotaibi A.A., Alabdali S.H., Zaalah A.A., Maghfuri M.W., Qirati N.H., Jandali Y.M., Almalki S.M. (2021). Acceptability of the COVID-19 Vaccine and Its Determinants among University Students in Saudi Arabia: A Cross-Sectional Study. Vaccines.

[53] European Medicines Agency EMA Considerations on COVID-19 Vaccine Approval. EMA/592928/2020. https://www.ema.europa.eu/en/documents/other/ema-considerations-covid-19-vaccine-approval_en.pdf.

