# Supplementary material for: Percentages of Vaccination Coverage Required to Establish Herd Immunity against SARS-CoV-2
Source: Vaccines (Basel). 2022 May 8;10(5):736. doi: 10.3390/vaccines10050736 (PMC9144560; doi:10.3390/vaccines10050736)
Supplement: Supplementary file 1 [file vaccines-10-00736-s001.zip › vaccines-1627158-supplementary.pdf]

Supplementary material

Table S1. Completed COVID-19 vaccination coverage in countries of the world (April 2022). Data from Our World in Data (https://ourworldindata.org/covid-vaccinations)

Table S2. Critical vaccination coverage (V_c) required to establish herd immunity against SARS-CoV-2 with reproductive numbers (R_o) from 1.1 to 10 by vaccination effectiveness

Table S3. Critical vaccination coverage (V_c) required to establish herd immunity against SARS-CoV-2 with different basic reproductive numbers (R_o) by COVID-19 vaccination effectiveness, with 10% of individuals protected and 9.8% infections among vaccinated individuals

Table S4. Critical vaccination coverage (V_c) required to establish herd immunity against SARS-CoV-2 with different basic reproductive numbers (R_o) by vaccine effectiveness, with 20% of individuals protected and 9.8% infections among vaccinated individuals

Table S1. Completed COVID-19 vaccination coverage in countries of the world (April 2022). Data from Our World in Data (https://ourworldindata.org/covid-vaccinations)

Country	%
Afghanistan	11.4
Albania	42.5
Algeria	13.7
Andorra	69.0
Angola	17.7
Antigua and Barbuda	62.5
Argentina	81.2
Armenia	33.0
Aruba	76.1
Australia	82.8
Austria	73.0
Azerbaijan	47.3
Bahamas	39.5
Bahrain	69.6
Bangladesh	69.0
Barbados	52.6
Belarus	58.3
Belgium	78.5
Belize	51.8
Benin	19.5
Bhutan	4.7
Bolivia	49.1
Bosnia and Herzegovina	
Botswana	53.9
Brazil	75.5
Brunei	91.8
Bulgaria	29.8
Burkina Faso	5.5
Burundi	0.1
Cambodia	83.1
Cameroon	3.9
Canada	82.0
Cape Verde	54.6
Central African Republic	18.3
Chad	4.5
Chile	90.6
China	86.1
Colombia	68.3
Comoros	33.9
Congo	12.0

Cook Islands	83.5
Costa Rica	78.0
Cote d'Ivoire	17.0
Croatia	54.9
Cuba	87.6
Curacao	59.8
Cyprus	
Czechia	64.0
Democratic Republic of Congo	0.6
Denmark	82.6
Djibouti	10.9
Dominica	41.6
Dominican Republic	54.3
Ecuador	77.2
Egypt	30.9
El Salvador	65.8
Equatorial Guinea	14.5
Estonia	63.6
Eswatini	27.2
Ethiopia	17.8
Fiji	69.6
Finland	77.7
France	77.8
Gabon	11.1
Gambia	12.8
Georgia	31.6
Germany	75.3
Ghana	16.0
Greece	73.3
Grenada	33.6
Guatemala	32.9
Guinea	18.4
Guinea-Bissau	16.9
Guyana	45.8
Haiti	1.0
Honduras	45.5
Hungary	64.2
Iceland	78.7
India	60.0
Indonesia	58.2
Iran	67.1
Iraq	17.6
Ireland	80.5
Israel	66.0
Italy	79.3

Jamaica	22.8
Japan	80.0
Kazakhstan	48.4
Kenya	14.9
Kiribati	45.6
Kosovo	46.1
Kuwait	75.9
Kyrgyzstan	18.9
Laos	62.5
Latvia	69.8
Lebanon	32.6
Lesotho	33.7
Liberia	20.9
Libya	16.3
Liechtenstein	69.0
Lithuania	69.6
Luxembourg	72.6
Madagascar	3.7
Malawi	4.4
Malaysia	78.9
Maldives	70.6
Mali	4.7
Malta	90.5
Mauritania	22.3
Mauritius	76.2
Mexico	61.2
Moldova	26.3
Monaco	
Mongolia	65.3
Montenegro	44.9
Morocco	62.6
Mozambique	41.1
Myanmar	40.2
Namibia	14.9
Nauru	70.6
Nepal	64.9
Netherlands	72.2
New Zealand	79.4
Nicaragua	65.6
Niger	6.2
Nigeria	4.5
Niue	87.8
North Macedonia	40.1
Northern Ireland	70.3
Norway	73.7
Oman	57.9

Pakistan	51.4
Panama	69.3
Papua New Guinea	2.8
Paraguay	46.1
Peru	78.4
Philippines	59.7
Poland	59.2
Portugal	92.6
Qatar	88.5
Romania	42.3
Russia	50.0
Rwanda	61.4
Saint Kitts and Nevis	
Saint Lucia	28.6
Saint Vincent and the Grenadines	27.1
Samoa	66.0
San Marino	69.4
Sao Tome and Principe	38.9
Saudi Arabia	69.6
Senegal	6.0
Serbia	47.6
Seychelles	81.0
Sierra Leone	13.8
Singapore	91.1
Slovakia	50.7
Slovenia	58.7
Solomon Islands	17.4
Somalia	8.3
South Africa	30.0
South Korea	86.8
South Sudan	4.2
Spain	86.1
Sri Lanka	67.1
Sudan	7.5
Suriname	0.1
Sweden	75.0
Switzerland	68.6
Syria	7.4
Taiwan	76.8
Tajikistan	49.8
Tanzania	5.0
Thailand	72.0
Timor	44.0
Togo	18.4
Tonga	65.8

Trinidad and Tobago	50.6
Tunisia	53.2
Turkey	62.3
Turkmenistan	
Tuvalu	
Uganda	17.0
Ukraine	
United Arab Emirates	96.3
United Kingdom	72.6
United States	65.7
Uruguay	81.9
Uzbekistan	41.8
Vanuatu	27.1
Venezuela	49.8
Vietnam	79.2
Yemen	1.3
Zambia	11.9
Zimbabwe	23.7
Regions of the World	
South America	73.2
Asia	67.7
Europe	65.3
Oceania	62.9
North America	62.7
Africa	15.4
World	58.2

Table S2. Critical vaccination coverage (V_c) required to establish herd immunity against SARS-CoV-2 with reproductive numbers (R_o) from 1.1 to 10 by vaccination effectiveness

R_o of SARS-CoV-2[c]	Critical vaccination coverage (%)[a] for Covid-19 vaccine effectiveness from 10% to 100%									
	10%	20%	30%	40%	50%	60%	70%	80%	90%	100%
1.1	90.9	45.5	30.3	22.7	18.2	15.2	13.0	11.4	10.1	9.1
1.25	–	100	66.7	50.0	40.0	33.3	28.6	25.0	22.2	20.0
1.5	–	–	–	83.3	66.7	55.6	47.6	41.7	37.0	33.3
1.75	–	–	–	–	85.7	71.4	61.2	53.6	47.6	42.9
2	–	–	–	–	100	83.3	71.4	62.5	55.6	50.0
2.25	–	–	–	–	–	92.6	79.4	69.4	61.7	55.6
2.5	–	–	–	–	–	100	85.7	75.0	66.7	60.0
2.75	–	–	–	–	–	–	90.9	79.5	70.7	63.6
3	–	–	–	–	–	–	95.2	83.3	74.1	66.7
3.25	–	–	–	–	–	–	98.9	86.5	76.9	69.2
3.5	–	–	–	–	–	–	–	89.3	79.4	71.4
3.75	–	–	–	–	–	–	–	91.7	81.5	73.3
4	–	–	–	–	–	–	–	93.8	83.3	75.0
4.25	–	–	–	–	–	–	–	95.6	85.0	76.5
4.5	–	–	–	–	–	–	–	97.2	86.4	77.8
4.75	–	–	–	–	–	–	–	98.7	87.7	78.9
5	–	–	–	–	–	–	–	100	88.9	80.0
6	–	–	–	–	–	–	–	–	92.6	83.3
7	–	–	–	–	–	–	–	–	95.2	85.7
8	–	–	–	–	–	–	–	–	97.2	87.5
9	–	–	–	–	–	–	–	–	98.8	88.9
10	–	–	–	–	–	–	–	–	100	90.0

a. Vc = Ic/Effectiveness. The critical vaccination coverage is not indicated (–) when herd immunity could not be established with 100% vaccination coverage
b. Ic = 1 – (1/R_o).
c. The basic reproduction number Ro indicates the average number of secondary cases generated per infected case in a completely susceptible population

Table S3. Critical vaccination coverage (V_c) required to establish herd immunity against SARS-CoV-2 with different basic reproductive numbers (R_o) by COVID-19 vaccination effectiveness, with 10% of individuals protected and 9.8% infections among vaccinated individuals

R_o of SARS-CoV[c]	Critical vaccination coverage (%)[a] for Covid-19 vaccine effectiveness from 10% to 100%									
	10%	20%	30%	40%	50%	60%	70%	80%	90%	100%
1.1	0	0	0	0	0	0	0	0	0	0
1.25	–	98.0	49.5	33.1	24.9	19.9	16.6	14.2	12.5	11.1
1.5	–	–	–	77.3	58.0	46.5	38.8	33.2	29.1	25.9
1.75	–	–	–	–	81.7	65.5	54.6	46.8	41.0	36.4
2	–	–	–	–	99.5	79.7	66.4	57.0	49.9	44.3
2.25	–	–	–	–	–	90.7	75.7	64.9	56.8	50.5
2.5	–	–	–	–	–	99.6	83.1	71.2	62.3	55.4
2.75	–	–	–	–	–	–	89.1	76.4	66.9	59.5
3	–	–	–	–	–	–	94.1	80.7	70.7	62.8
3.25	–	–	–	–	–	–	98.4	84.4	73.9	65.7
3.5	–	–	–	–	–	–	–	87.5	76.6	68.1
3.75	–	–	–	–	–	–	–	90.2	79.0	70.2
4	–	–	–	–	–	–	–	92.6	81.0	72.1
4.25	–	–	–	–	–	–	–	94.7	82.9	73.7
4.5	–	–	–	–	–	–	–	96.5	84.5	75.1
4.75	–	–	–	–	–	–	–	98.2	86.0	76.4
5	–	–	–	–	–	–	–	99.7	87.3	77.6
6	–	–	–	–	–	–	–	–	91.4	81.3
7	–	–	–	–	–	–	–	–	94.4	83.9
8	–	–	–	–	–	–	–	–	96.6	85.9
9	–	–	–	–	–	–	–	–	98.4	87.5
10	–	–	–	–	–	–	–	–	99.8	88.7

a. $V_c = I_c$/Effectiveness. $I_c = 1 - (1/R_o)$. The critical vaccination coverage is not indicated (–) when herd immunity could not be established with 100% vaccination coverage

b. The basic reproduction number R_o indicates the average number of secondary cases generated per infected case in a completely susceptible population

Table S4. Critical vaccination coverage (V_c) required to establish herd immunity against SARS-CoV-2 with different basic reproductive numbers (R_o) by vaccine effectiveness, with 20% of individuals protected and 9.8% infections among vaccinated individuals

R_o of SARS-CoV[c]	Critical vaccination coverage (%)[a] for Covid-19 vaccine effectiveness from 10% to 100%									
	10%	20%	30%	40%	50%	60%	70%	80%	90%	100%
1.1	0	0	0	0	0	0	0	0	0	0
1.25	0.0	0.0	0.0	0.0	0.0	0.0	0.0	0.0	0.0	0.0
1.5	–	–	66.0	44.2	33.2	26.6	22.1	19.0	16.6	14.8
1.75	–	–	–	75.7	56.9	45.5	38.0	32.6	28.5	25.3
2	–	–	–	99.3	74.6	59.8	49.8	42.7	37.4	33.3
2.25	–	–	–	–	88.4	70.8	59.1	50.6	44.3	39.4
2.5	–	–	–	–	99.5	79.7	66.4	57.0	49.9	44.3
2.75	–	–	–	–	–	86.9	72.5	62.2	54.4	48.4
3	–	–	–	–	–	93.0	77.5	66.5	58.2	51.7
3.25	–	–	–	–	–	98.1	81.8	70.1	61.4	54.6
3.5	–	–	–	–	–	–	85.4	73.3	64.1	57.0
3.75	–	–	–	–	–	–	88.6	76.0	66.5	59.1
4	–	–	–	–	–	–	91.4	78.3	68.6	61.0
4.25	–	–	–	–	–	–	93.8	80.4	70.4	62.6
4.5	–	–	–	–	–	–	96.0	82.3	72.0	64.1
4.75	–	–	–	–	–	–	97.9	84.0	73.5	65.4
5	–	–	–	–	–	–	99.7	85.5	74.8	66.5
6	–	–	–	–	–	–	–	90.2	79.0	70.2
7	–	–	–	–	–	–	–	93.6	81.9	72.9
8	–	–	–	–	–	–	–	96.2	84.2	74.8
9	–	–	–	–	–	–	–	98.1	85.9	76.4
10	–	–	–	–	–	–	–	99.7	87.3	77.6

a. $V_c = I_c$/Effectiveness. $I_c = 1 - (1/R_o)$. The critical vaccination coverage is not indicated (−) when herd immunity could not be established with 100% vaccination coverage
b. The basic reproduction number R_o indicates the average number of secondary cases generated per infected case in a completely susceptible population
